# Tackling TKI resistance in AML: A commentary on “Inhibition of BCL2A1 by STAT5 inactivation overcomes resistance to targeted therapies of FLT3-ITD/D835 mutant AML.” by Yamatani et al.

**DOI:** 10.1016/j.tranon.2022.101394

**Published:** 2022-03-14

**Authors:** Özden Hatırnaz Ng, Ahmet Emre Eşkazan

**Affiliations:** a Department of Medical Biology, School of Medicine, Acıbadem University, Istanbul, Turkey; b Department of Internal Medicine, Division of Hematology, Cerrahpaşa Faculty of Medicine, Istanbul University-Cerrahpaşa, Fatih, Istanbul, Turkey

**Keywords:** Acute myeloid leukemia, AML, *FLT3*, *FLT3-ITD/D835*, *BCL2A1*, Resistance

Acute myeloid leukemia (AML) is a heterogenous malignancy of immature myeloid cells developed due to uncontrolled proliferation. The underlying mechanism can partially be explained by variable number of chromosomal abnormalities, gene variations, epigenetic modifications and deregulated gene expressions. Other than cytogenetic abnormalities, driver and passenger variations have been described in more than 95% of AML patients and it is reported that an AML genome can bear more than ten somatic genetic variations [1]. This high heterogeneity also brings a challenge into the treatment of AML. The 5-year overall survival estimation in AML is around 24% [2], 50%−60% of the AML patients relapse, and this rate decreases down to 10% in patients with advanced age (> 65 years) [3].

With the recent advancements in high throughput technologies, numerous molecular markers have been described in AML and based on these molecular markers the risk groups were released by the European LeukemiaNet (ELN) [4], which covers recurrent genetic variations in genes like *FMS-like tyrosine kinase 3* (*FLT3), Nucleophosmin 1 (NPM1)* and *CCAAT Enhancer Binding Protein Alpha* (*CEBPA).*The discovery of new genetic biomarkers provided a high improvement in the targeted therapies for AML cases as well. *FLT3*-targeted tyrosine kinase inhibitors (TKIs) can be given as an effective example in the treatment of AML. FLT3, is a transmembrane tyrosine kinase, which is expressed both in myeloid and lymphoid cells. The *FLT3* variations are found in around 30% of AML patients either as internal tandem repeats (ITD, 20–25%) or affecting tyrosine kinase domain (TDK, 7–10%) [5]. Both variations constitutively activate the FLT3, which leads to the proliferation and survival of AML cells. *FLT3* variations are counted as driver mutations and studies report that the patients with *FLT3* variations showed low prognosis, unfavorable outcome and lower overall survival than the patients without *FLT3* variations.

In the last 5 years, the therapeutic armamentarium of AML expanded extensively, and there are many options available both in the upfront and salvage settings [6], including those that target FLT3*.* FLT3 TKIs are classified either according to their mechanism of action (Type I and Type II) or in order of their development (First-generation and Second-generation). First-generation inhibitors were targeting both ITD and TDK variations but therapy-related variations in *FLT3* lead to therapy resistance. The second-generation TKIs were developed to inactive the FLT3 conformation and may overcome resistance related variations. Gilteritinib, is a type I and second-generation inhibitor, which is effective against both ITD and mutant TKD [7]. Gilterinitib has been approved by the United States Food and Drug Administration (FDA) in the management of AML, following the promising results of the open-label, multicenter, randomized phase III ADMIRAL study (NCT02421939), where gilteritinib was compared with salvage chemotherapy in relapsed/refractory *FLT3*-mutated AML patients [8]. Despite all promising achievements, therapy-related resistance is still an important obstacle on the way to a successful treatment.

The detailed understanding of the FLT3 mechanism also allowed development of different agents against the different points of FLT3 domains. In the study of Yamatani et al [9]., the authors aimed to overcome therapy resistance due to a specific variation, *FLT3*-ITD/D835. The findings of the study lead the authors to a new mechanism that is regulated by the overexpression of *BCL2A1* and the authors suggested that reversing this over-expression may increase the therapy success and *BCL2A1* can be a novel target in refractory AML cases. They first investigate the transcriptomic and mutation profiles of primary AML patients’ samples with *FLT3*-ITD or *FLT3*-ITD/D835 and found that *FLT3*-ITD/D835 cases were bearing co-mutations more often than *FLT3*/ITDs and showing higher *BCL2A1* RNA expression. They also overexpressed *BCL2A1* by a lentiviral vector in cell lines carrying *FLT3*-ITD variation and treated them with quizartinib, which is a second-generation TKI used in the treatment of *FLT3*-ITD cases [10]. The transfected cells were able to escape from apoptosis caused by the drug treatment whereas non-transfected cells were more sensitive to quizartinib. They also evaluated the combined treatment of quizartinib and venetoclax on cell lines bearing *FLT3*-ITD and *FLT3*-ITD/D835 variations. Venetoclax targets *BCL2* and leads AML cells to apoptosis without causing thrombocytopenia [11]. The cells with *FLT3*-ITD/D835 were less sensitive to quizartinib and venetoclax alone. On the other hand, although the cells with *FLT3*-ITD showed a significant decrease to a combined treatment of quizartinib and venetoclax, no significant difference was detected in *FLT3*-ITD/D835 cells. They also combined the venetoclax with gilterinitib, and this time they were able to show that the proliferation was inhibited in both cell types (*FLT3*-ITD and *FLT3*-ITD/D8935) for gilterinitib and the combination of venetoclax and gilterinitib. The following experiments showed that the solo effect of gilterinitib blocks only STAT5-phophorylation and the combining venetoclax not only decreases the BLC2A1 but also MCL-1 protein expression. STAT5, is a transcription factor that regulates *BCL2* [12]. *BCL-2* and *MCL-1* are apoptotic suppressors, and their downregulation leads the malignant cells to apoptosis [11]. Last but not least they also treated the cell lines with a molecule, CP-0610, that is a *BCL2A1* inhibitor and the molecule decreased the cell proliferation in a dosage dependent manner and the *BCL2A1* expressions were downregulated. In summary, the authors successfully overcome the *FLT3*-ITD/D835 variation by inhibiting *BCL2A1* via STAT5 pathway and they suggested that *BCL2A1* specific inhibitors may be used as alternative treatment for cases bearing variations in more than one gene.

AML treatment is still challenging with its heterogeneous and dynamic genetic background. Advances in the molecular technologies, defining mechanisms of AML development and/or resistance, will allow more agents to come to the field and every effort on describing new targetable molecules will improve the treatment outcome. Similar studies like the work by Yamanati and colleagues [9] can create basis for new studies not only on AML treatment, but also on different hematologic malignancies with complex mechanisms. Describing new targets may also lead to new targeted therapies to be developed like small oligonucleotides and chimeric antigen receptors. Also following therapy response by minimal residual disease measurements will also improve the treatment success. There is still a long path to take in the treatment of AML, but each promising step will be beneficial for the patients.

## Funding statement

This research did not receive any specific grant from funding agencies in the public, commercial, or not-for-profit sectors.

## CRediT authorship contribution statement

**Özden Hatırnaz Ng:** Conceptualization, Methodology, Investigation, Writing – original draft. **Ahmet Emre Eşkazan:** Conceptualization, Methodology, Supervision, Writing – review & editing.

## Declaration of Competing Interest

The authors declare that they have no known competing financial interests or personal relationships that could have appeared to influence the work reported in this paper.
